# A2B-COVID: A Tool for Rapidly Evaluating Potential SARS-CoV-2 Transmission Events

**DOI:** 10.1093/molbev/msac025

**Published:** 2022-02-02

**Authors:** Christopher J R Illingworth, William L Hamilton, Christopher Jackson, Ben Warne, Ashley Popay, Luke Meredith, Myra Hosmillo, Aminu Jahun, Tom Fieldman, Matthew Routledge, Charlotte J Houldcroft, Laura Caller, Sarah Caddy, Anna Yakovleva, Grant Hall, Fahad A Khokhar, Theresa Feltwell, Malte L Pinckert, Iliana Georgana, Yasmin Chaudhry, Martin Curran, Surendra Parmar, Dominic Sparkes, Lucy Rivett, Nick K Jones, Sushmita Sridhar, Sally Forrest, Tom Dymond, Kayleigh Grainger, Chris Workman, Effrossyni Gkrania-Klotsas, Nicholas M Brown, Michael P Weekes, Stephen Baker, Sharon J Peacock, Theodore Gouliouris, Ian Goodfellow, Daniela De Angelis, M Estée Török

**Affiliations:** 1 MRC-University of Glasgow Centre for Virus Research, Glasgow, United Kingdom; 2 MRC Biostatistics Unit, University of Cambridge, Cambridge, United Kingdom; 3 Department of Applied Mathematics and Theoretical Physics, University of Cambridge, Cambridge, United Kingdom; 4 Institut für Biologische Physik, Universität zu Köln, Köln, Germany; 5 Department of Medicine, University of Cambridge, Cambridge, United Kingdom; 6 Cambridge University Hospitals NHS Foundation Trust, Cambridge, United Kingdom; 7 Public Health England Field Epidemiology Unit, Cambridge Institute of Public Health, Cambridge, United Kingdom; 8 Department of Pathology, Division of Virology, University of Cambridge, Cambridge, United Kingdom; 9 Clinical Microbiology and Public Health Laboratory, Cambridge University Hospitals NHS Foundation Trust, Cambridge, United Kingdom; 10 Francis Crick Institute, London, United Kingdom; 11 Cambridge Institute for Therapeutic Immunology and Infectious Disease, Jeffrey Cheah Biomedical Centre, Cambridge, United Kingdom; 12 Wellcome Sanger Institute, Hinxton, United Kingdom; 13 MRC Epidemiology Unit, University of Cambridge, Level 3 Institute of Metabolic Science, Cambridge, United Kingdom; 14 School of Clinical Medicine, University of Cambridge, Cambridge, United Kingdom; 15 Public Health England, National Infection Service, London, United Kingdom

**Keywords:** SARS-CoV-2, transmission, evolution, hospital

## Abstract

Identifying linked cases of infection is a critical component of the public health response to viral infectious diseases. In a clinical context, there is a need to make rapid assessments of whether cases of infection have arrived independently onto a ward, or are potentially linked via direct transmission. Viral genome sequence data are of great value in making these assessments, but are often not the only form of data available. Here, we describe A2B-COVID, a method for the rapid identification of potentially linked cases of COVID-19 infection designed for clinical settings. Our method combines knowledge about infection dynamics, data describing the movements of individuals, and evolutionary analysis of genome sequences to assess whether data collected from cases of infection are consistent or inconsistent with linkage via direct transmission. A retrospective analysis of data from two wards at Cambridge University Hospitals NHS Foundation Trust during the first wave of the pandemic showed qualitatively different patterns of linkage between cases on designated COVID-19 and non-COVID-19 wards. The subsequent real-time application of our method to data from the second epidemic wave highlights its value for monitoring cases of infection in a clinical context.

## Introduction

The COVID-19 pandemic remains a global public health priority (Andersen et al. 2020; Dong et al. 2020). Understanding the nature of viral transmission and identifying linked cases are both critical to inform and optimize infection prevention and control (IPC) strategies. This is especially important in healthcare settings, where SARS-CoV-2 can spread rapidly between patients and staff via asymptomatic or pauci-symptomatic intermediates, and vulnerable patients may be susceptible to severe disease. Hospital-acquired COVID-19 has been associated with substantial morbidity and mortality, and with the emergence and spread of new variants with greater infectivity. Reducing SARS-CoV-2 transmission within hospitals is of pressing concern (Richterman et al. 2020; Wake et al. 2020; Read et al. 2021; Rickman et al. 2021).

Viral genome sequencing provides one strategy for identifying possible clusters of transmission. Viral populations accumulate genetic variation over time through the evolutionary processes of mutation, selection, and genetic drift. If viral sequences from two individuals are more genetically different from each other than might be expected given a model of sequence evolution in transmission, then the occurrence of direct transmission between the two becomes less likely. A range of approaches for identifying linked infection clusters using genomic data have been suggested (Brenner et al. 2011; Ragonnet-Cronin et al. 2013; Gire et al. 2014; Jacka et al. 2014; McCloskey and Poon 2017). However, similar genomes do not necessarily indicate epidemiological linkage. Putative clusters identified through genomics must be integrated with epidemiological data to obtain a robust interpretation of events.

Several studies have used genomics to investigate SARS-CoV-2 transmission in hospitals, identifying clusters of potential hospital-based transmission (Lucey et al. 2020; Safdar et al. 2020; Ellingford et al. 2021; Frampton et al. 2021). When genomic analyses are made available rapidly they can help to inform real-time decision-making, by clinicians, IPC, and hospital management (Meredith et al. 2020; Hamilton, Fieldman, et al. 2021; Stirrup et al. 2021). Major challenges to applying SARS-CoV-2 genomics prospectively in a hospital context include the time taken from sample to sequence to analysis, the integration of data from multiple sources into a coherent analysis, and the presentation of results in a manner that can easily be understood.

Here, we address these challenges with the software tool A2B-COVID. A2B-COVID is designed to provide rapidly interpretable information to clinical staff who may lack specific expertise in genetics or evolutionary biology, to enable accurate and intuitive decisions to be made (Croskerry 2009). Within this context, we reduce the problem of identifying cases of viral transmission to a simple question, asking, on the basis of data from two individuals A and B, whether or not these data are consistent with an underlying hypothesis of direct SARS-CoV-2 transmission from A to B. Our method combines genome sequence data, information about the location of individuals, and knowledge of SARS-CoV-2 transmission dynamics to produce a clear and interpretable output. Although the combination of different data sources requires a nontrivial analysis, the ease of use of our approach has particular merit in situations where public health and IPC resources may be stretched. When applied in a clinical context, outputs from our method can be followed up with more detailed analysis by an IPC team, who may have access to information that is less easily quantifiable or available for analysis. While sequence and symptom data can never definitively prove that one person infected another, our method focuses the attention of busy clinicians upon potential cases of nosocomial transmission.

We here present and discuss outputs from the retrospective application of our method to simulated and clinical data, and report the prospective use of our method during the second wave of the UK pandemic in Cambridge University Hospitals NHS Foundation Trust (CUH). We describe how the prospective application of our method helped to inform hospital policy on personal protective equipment for staff working on COVID-19 wards.

## Results

**Fig. 1.**
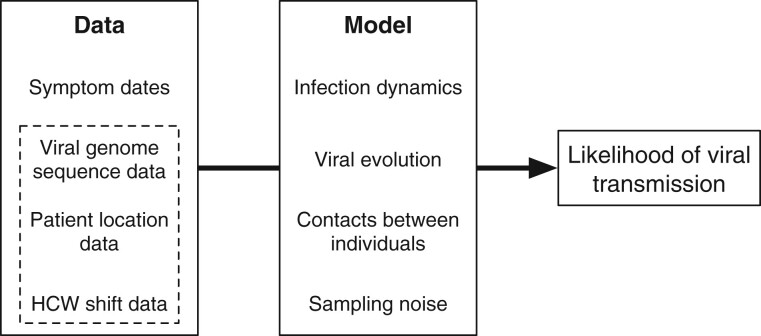
Overview of our method. Our approach estimates the likelihood that transmission could have occurred between pairs of individuals. The model takes as input dates on which individuals became symptomatic for COVID-19 infection. Further data which can be considered includes viral genome sequence data, and time-resolved location data for each individual. Our model combines details of COVID-19 infection dynamics with a model of viral evolution, information about potential contacts between individuals, and measurement error in the sequence data. Increasing amounts of data provide increasing amounts of resolution about the potential for viral transmission.

**Fig. 2.**
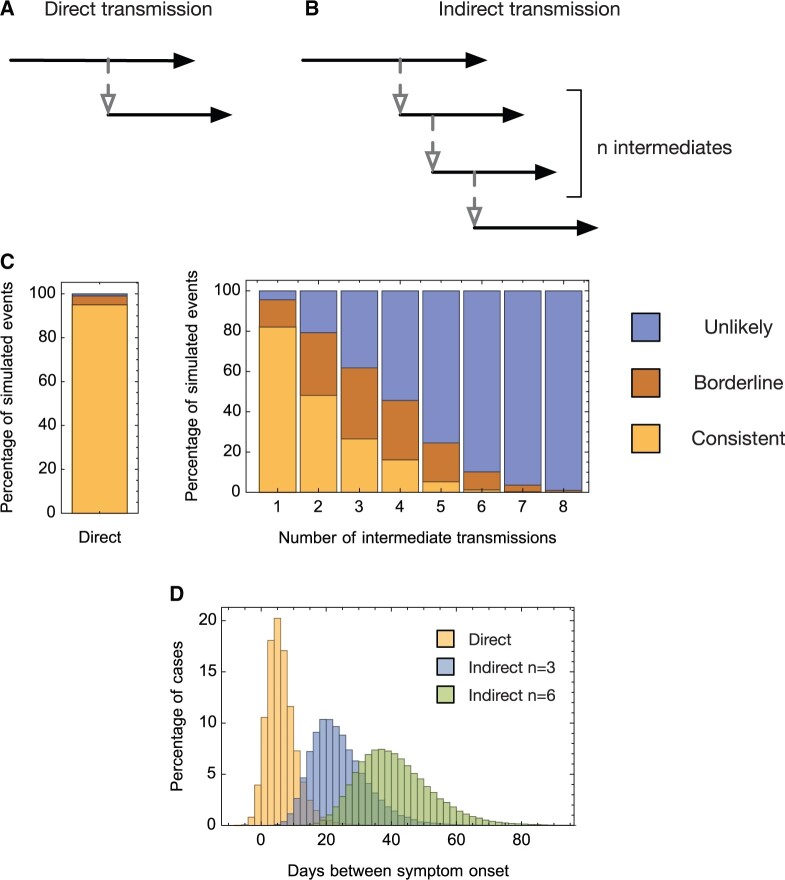
Analysis of simulated data. Simulations were performed describing (*A*) direct and (*B*) indirect transmission events. (*C*) Results of analyses using A2B-COVID. 95% of data sets from direct transmission events were identified as consistent with direct transmission, as designed. Data from increasingly separated pairs of individuals showed decreasingly fewer events identified as consistent with direct transmission. (*D*) Days between symptom onsets for selected simulated data sets. The low mean and high variance in the time between symptom dates leads to a tradeoff between the recall and the precision of our method.

We first demonstrated our method by application to simulated data describing direct and indirect transmission events. Our method combines multiple types of data, using the information available to identify potentially linked cases of infection (fig. 1). For the purpose of method testing, we considered a series of potential relationships between infections, generating 10^5^ simulated events for each, and recording the times of symptom onset of two individuals A and B, alongside simulated times at which whole viral genomes were collected, and numbers of distinct variants detected in these genome sequences (fig. 2*A* and *B*). A simple model of location was applied, assuming individuals to have a one in four chance of being in contact on any given day. Our method classifies data as being either “consistent” with direct transmission, “unlikely” to have been observed from a direct transmission event, or “borderline,” between these two cases; data from simulated transmission events was classified in this manner.

Our method successfully identified direct transmission events with the desired level of recall. Applied to data from simulations of direct transmission events, our model identified 95% of data sets as being “consistent” with a hypothesis of direct transmission (fig. 2*C*), with a further 4.1% identified as “borderline.” Applied to data describing indirect transmission relationships between A and B, data were sometimes also found to be consistent with direct transmission. For example, applied to data from which a single individual separated A and B, 82.1% of data sets were judged “consistent” with transmission; this figure fell to 26.5% of data sets when three individuals separated A and B, and 1% of data sets when six individuals separated A and B. The reason for this identification is evident from the distribution of times between symptom onset in A and B generated by different transmission relationships (fig. 2*D*); whereas the mean difference in times of symptom onset is relatively small, at around 5 days, the variance in this difference is large, so that events separated by multiple transmissions can be consistent with the occurrence of direct transmission.

Our application to simulated data showed that the performance of our method was consistent with our requirements. As discussed below, our method could be tuned to increase or decrease the recall of genuine cases of direct transmission with the consequence of a correspondingly increased or decreased identification of indirectly related cases.

Considering the application of our method to clinical data, we first evaluated measurement error in the sequencing pipeline used to generate virus consensus sequences. Multiple studies have considered the problem of noise in genome sequence data, particularly with regard to identifying variant frequencies (Beerenwinkel and Zagordi 2011; Laehnemann et al. 2016; Illingworth et al. 2017; Sandmann et al. 2017). Using data from cases in which more than one sample was collected from the same host, we inferred a mean error rate of approximately 0.207 nucleotide errors per sequence (supplementary fig. S1, Supplementary Material online). With an expected generation time for transmission of 5.7 days, we note that the measurement error is close to the expected amount of within-host evolution in a transmission event (supplementary fig. S2, Supplementary Material online).

The retrospective application of our model to data from two wards within CUH showed its ability to provide a useful shortlisting of potential transmission events. Data from two wards, here labeled X and Y, had been collected during the first wave of infection (March to June 2020). Ward X was a “green” ward, used for patients considered to be free from COVID-19 infection, whereas ward Y was a “red” ward, designated for the treatment of patients with COVID-19 infection, where multiple cases of infection in healthcare workers (HCWs) had been identified. Information collected for these individuals included viral genome sequence data from diagnostic swabs, dates of symptom onset, and dates on which individuals were present on the wards in question.

**Fig. 3.**
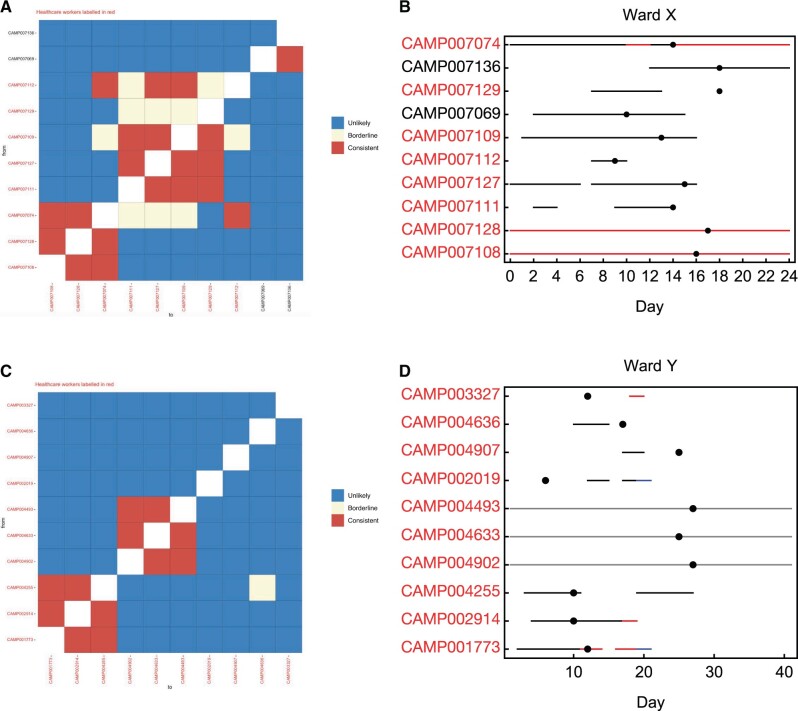
Analysis of the full data sets collected from wards X and Y. (*A*) Output from the A2B-COVID package given data from ward X. The plot shows potential links between cases, assessed in a pairwise fashion between potential donors (rows) and recipients (columns). Identifiers of individuals are colored in either black (patients) or red (HCWs). Squares in the grid indicate that transmission from one individual to another is consistent with our model (red), borderline (yellow), or unlikely (blue). (*B*) Locations of individuals linked to the ward X outbreak. Black lines indicate presence on ward X. Red lines indicate known household contacts between three individuals. Dots show times at which individuals first reported symptoms. (*C*) Output from the A2B-COVID package given data from ward Y. (*D*) Locations of individuals linked to the ward Y outbreak. Black lines indicate presence on ward Y. Red and blue lines show presence in locations other than ward Y.

Outputs from our method show a meaningful identification of potential transmission events on each ward (fig. 3). On Ward X, a total of 28 transmission events out of a total of 90 were identified as consistent with direct transmission, with 11 out of 90 possible events identified on Ward Y. We note that outputs from our method are asymmetrical. For example, the data were consistent with transmission from individual 7,069 to individual 7,074 having occurred, but not consistent with a transmission event from 7,074 to 7,069, which was ruled unlikely. This result can be explained by individual 7,069 reporting symptoms 4 days before individual 7,074; the relative timing of symptoms provides information on the likely direction of transmission. Our data suggest that infections in Ward X could potentially constitute a single outbreak, with a single introduction onto the ward leading to subsequent transmission to patients and HCWs. In contrast, the data from Ward Y suggest that the majority of cases were independent of one another, with two clusters of three infections among HCWs being identified. This pattern fits the designation of the ward as a red ward, where new COVID-19 patients were routinely admitted.

Testing suggested that both location data and sequencing data were of value in assessing potential transmission events. In our model, location data constrain the potential for transmission; two individuals could only transmit to one another if they were in the same place at the same time. In the absence of location data, individuals were assumed to be colocated (see Materials and Methods for compete details); a reanalysis of the cases without location data, or without sequence data, each showed poorer discrimination (supplementary figs. S3 and S4, Supplementary Material online). For example, in the absence of sequence data, two pairs on ward X were assessed as being consistent with transmission where with the addition of sequence data these events were unlikely, whereas one pair was assessed as unlikely when sequence data showed it to be consistent. On ward Y, nine pairs were assessed as being consistent with transmission in the absence of sequence where the addition of sequence data these events were unlikely, whereas five pairs were assessed as unlikely when sequence data showed it to be consistent; sequence data are important to our calculations. A simple sequence-based test, using a cutoff of two nucleotide differences between sequences, identified multiple pairs of cases as being consistent with transmission which our regular analysis identified as clearly unrelated (supplementary fig. S5, Supplementary Material online). Further tests indicated that neglecting noise in genome sequencing also affected our calculations. Either increasing or decreasing this parameter from the inferred value led to changes in the categorizations of some events (supplementary fig. S6, Supplementary Material online).

**Fig. 4.**
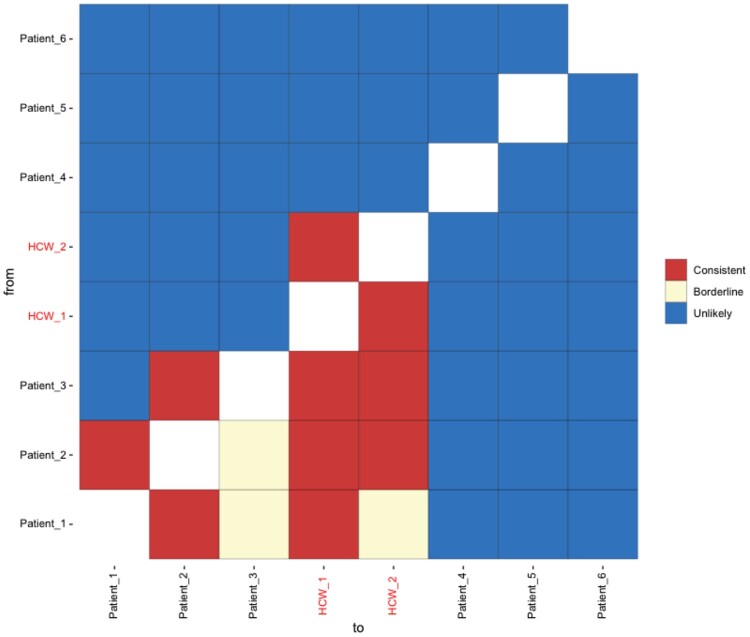
Output analysis from the real-time application to clinical wards. Output from the A2B-COVID app applied to data from a COVID-19 ward during the second wave of infection in the UK. Data from the patients 1, 2, and 3 is consistent with the direct infection of the health care workers HCW_1 and HCW_2.

Having tested our method, A2B-COVID was used for the real time analysis of data by clinicians at CUH during the second wave of infection (October 2020 to January 2021). The output from A2B-COVID contributed to changes in clinical practice in the hospital, demonstrating the value of real-time genome sequence analysis in this context (Hamilton, Fieldman, et al. 2021). Figure 4 shows the output from A2B-COVID describing cases from a ward (“Ward Z”) for confirmed COVID-19 patients and two staff members who developed COVID-19 while working on the ward (HCW1 and HCW2), during the period of implementation. Of note, multiple links were observed whereby the HCWs could potentially have been infected by patients 1, 2, or 3. Electronic medical records for these patients were reviewed for evidence of direct contact with the HCWs, to further assess for epidemiological evidence of transmission. HCW1 directly cared for patient 2 and documented in their medical notes within the first 2 days of patient 2’s first positive test, and HCW1 developed symptoms 2–4 days later. HCW2 directly cared for patients 1 and 3 and documented in their medical notes 7–8 days prior to HCW2 developing symptoms. Patients 1 and 3 were around days 2 and 7 post onset at the time HCW2 documented in their medical notes, respectively. Viral sequences collected from patients 4 and 5 differed by a single shared SNP from the other sequences, which were otherwise identical. These data were consistent with SARS-CoV-2 transmission from patients to HCWs working on the COVID-19 ward, though we note that it does not prove that transmission did take place; the staff could potentially have been infected from other sources including outside of the hospital. These findings were presented at the CUH COVID-19 infection control meeting in January 2021. Using the precautionary principle, this evidence supported the decision (taken in late December 2020) to provide higher-grade (FFP3) respiratory protection, as opposed to fluid-resistant surgical masks, for all staff on COVID-19 wards. A subsequent study showed that this use of FFP3 masks significantly reduced the rate of ward-based infection among HCWs (Ferris et al. 2021). Our method thus has demonstrated value within a healthcare setting.

**Fig. 5.**
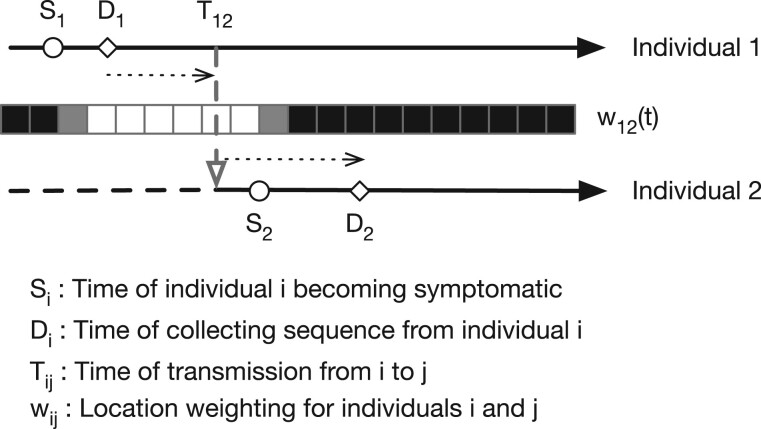
Notation used in our method. An overview of our model for transmission events is shown in figure 3. We divide time into discrete days. For the individual A, we denote by *S*_A_ the date at which that individual became symptomatic, and by *D*_A_ the date at which a sample of viruses were collected for genome sequencing. For each pair of individuals A and B, we denote by *w*_AB_(*t*) the probability that A and B were colocated on day *t*. Within our model, we assume that dates of sample collection are known, whereas times of symptom onset are known or estimated. Using these data, in combination with viral sequence data, we calculate a statistic describing the consistency of the data with individual A having infected individual B on any given day *T*. Summing this statistic across *T*, we obtain an estimate of the consistency of our data with transmission having occurred between the two individuals.

Although our method is designed as a tool for rapid analysis, flagging up potential cases of direct nosocomial viral transmission, it has the potential to be used as a first step in more detailed analyses of data. An extension of this framework inferring networks of transmission events has been used to assess levels of transmission between health care workers and patients, and to identify patterns of SARS-CoV-2 superspreading in a clinical context (Illingworth et al. 2021).

## Discussion

We have here described a tool for rapidly identifying potential cases of direct transmission between pairs of individuals, via a model utilizing the dynamics of SARS-CoV-2 infection, data describing the colocation of individuals, and genome sequence data collected during infection. In illustrative applications of our method, we analyzed data from wards in a large acute NHS hospital in the UK, identifying cases where the data were consistent with viral transmission occurring between either patients or HCWs on the ward. Our method incorporates data from multiple sources to present an easily interpretable map of potentially linked cases of infection. We believe that A2B-COVID is likely to be valuable in the initial assessment by health care workers of potential cases of transmission, highlighting pairs or clusters of individuals for further epidemiological assessment, and allowing for a more strategic deployment of resources for outbreak investigation and targeted interventions. This was particularly important during a period of high COVID-19 transmission in the UK, termed the “second wave” (October 2020 to February 2021 [Hamilton, Fieldman, et al. 2021]), before COVID-19 vaccination had been widely deployed and when the hospital infection control team came under intense pressure. Applied prospectively in a clinical setting during this period, the A2B-COVID tool provided results which helped to focus further investigation of potential HCW infections on a COVID-19 ward, contributing in real-time to hospital infection control policy decision-making.

Our method brings together a variety of data, combining an evolutionary model for the analysis of sequence data with location information and details of the dynamics of viral infection. In contrast to standard phylogenetic approaches to sequence data, our model explicitly accounts for noise in the generation of a viral consensus sequence; using within-host data we identified a magnitude of error of a fraction of one nucleotide per genome. In rapidly evolving viruses for which transmissions are separated by longer periods of time, the within-host evolution of viral populations is likely to overwhelm the effect of noise in the sequencing process. However, for cases of acute infection, separated by only a few days, the extent of noise may be close to the expected evolutionary change in the population, making it an important consideration.

Our model has a range of features specifically tailoring it to the real-time analysis of data in a hospital context during an outbreak of a rapidly spreading viral disease. Our method is designed for simplicity both in being easy to use and in producing an interpretable output with minimal computational requirements. It can tolerate a range of data inputs, from very basic (symptom onset and/or sample collection dates) to genome sequence data and information on patient and HCW colocations.

We note that the question addressed by our model, of the consistency of data from two individuals with direct transmission, is distinct from an estimation of the probability that A infected B. In a clinical context, it provides a first step toward further epidemiological investigation, which could consider data beyond that included in the model (e.g., measuring the locations of patients at a higher resolution than ward level, and the extent of contacts between health care workers and patients in a more precise manner than assessing who was on a given ward each day). Even with detailed retrospective epidemiological investigation it may be impossible to know for certain whether a specific transmission event occurred. However, each additional form of data supplied to our model contributes information to the output.

Our results highlight a challenge in the use or nonuse of location data in identifying potential transmission events. Our method optionally makes use of location data, setting the colocation of individuals as a necessary condition for transmission to occur on a particular day. Although accurate location data can exclude multiple cases where transmission cannot have occurred, incomplete location data can mean that genuine cases of transmission are excluded. In a hospital setting, this applies more to HCWs than to patients. Although patients are unlikely to be highly mobile, HCWs move around the hospital outside of their shifts. Unless explicitly recorded, off-ward contacts between HCWs may go unrecorded. Location data thus represent a powerful data set in refining the potential for transmission, but one which, if used without caution, may lead to the false exclusion of real connections between individuals.

We acknowledge several limitations of our method. Firstly, it deals with consensus viral sequences rather than deep sequence data. Where available, detailed measurements of within-host viral diversity may lead to an improved picture of relationships between cases of viral infection. Second, our tool analyses transmission relationships in a pairwise manner; whereas distinguishing plausible from implausible links between cases of infection, it does not attempt to infer a complete reconstruction of a transmission network. Third, unobserved cases of infection are not considered, evaluating only the question of potential transmission between known individuals. Fourth, the model uses parameters that are themselves derived from limited data. By default, our model parameters were setup to describe the original pandemic strain of SARS-CoV-2, though an option to use parameters derived for the Delta variant has now been included. Fifth, in so far as our model uses symptom onset dates we note that these statistics may be vulnerable to subjectivity or poor recall on the part of individuals reporting symptoms. Infections in vaccinated individuals may have a higher probability of being asymptomatic, therefore evading detection (Tang et al. 2021) Finally, we reiterate that the model must be properly understood for correct clinical interpretation; data being “consistent” with direct transmission does not imply that transmission did indeed take place. Although our software can provide valuable insights, it does not automate the process of a full epidemiological investigation.

We note that our method may have application to data from care homes, households, and other confined settings where a number of infections may be linked with one another (Aggarwal et al. 2022). Given appropriate changes to model parameters our method could be applied to other viruses for which healthcare transmission may be a concern (Sukhrie et al. 2012; Houldcroft et al. 2018; Godoy et al. 2020; Baller et al. 2021). However, we believe that a key application of our method will be investigating the nosocomial transmission of SARS-CoV-2. Within a hospital, potential cases of transmission may be obscured by a large number of cases of community-acquired infection. In a busy clinical setting, our tool has the ability to rapidly separate potentially linked cases from those which are likely to be unlinked. In this way we allow investigative efforts and epidemiological followup to be focused more precisely, concentrating effort on cases where transmission is a real possibility.

## Materials and Methods

### Study Setting, Participants, and Data Collection

This study was conducted at CUH, a secondary and tertiary referral center in the East of England (EoE). SARS-CoV-2 positive cases tested at the onsite Clinical Microbiology and Public Health Laboratory (CMPHL) were identified prospectively for genome sequencing as part of the COG-UK Consortium, as described in previous publications (Meredith et al. 2020; Hamilton, Fieldman, et al. 2021; Hamilton, Tonkin-Hill, et al. 2021). The CMPHL tests SARS-CoV-2 samples submitted from over 30 organizations across the EoE region and samples from CUH. The majority of samples were tested using an inhouse validated quantitative Reverse Transcription Polymerase Chain Reaction (qRT-PCR) assay targeting the SARS-CoV-2 RdRp genes (Meredith et al. 2020), with more recent samples tested using the Hologic Panther platform (Sridhar et al. 2020). Patient metadata were accessed via the electronic healthcare record system (Epic Systems, Verona, WI). Metadata collected included patient’s demographic information, duration of symptoms, sample collection date, and location (ward and hospital). Patients and samples were assigned unique anonymized study codes. Metadata manipulations were performed using the R programming language and the *tidyverse* packages installed on CUH Trust computers (as in Meredith et al. [2020], Hamilton, Fieldman, et al. [2021], and Hamilton, Tonkin-Hill, et al. [2021]). The outbreaks for wards X and Y occurred during the COVID-19 “first wave” (March–June 2020) and were investigated using *A2B-COVID* retrospectively. Ward Z was investigated in “real-time” during the COVID-19 “second wave” (October 2020–February 2021) (exact dates are not given to protect patient anonymity).

### Sample Sequencing

All samples collected at CUH and a randomized selection of samples from the EoE region were selected for nanopore sequencing onsite in the Division of Virology, Department of Pathology, University of Cambridge. This enabled us to rapidly investigate suspected hospital-acquired infections at CUH, as previously described (Meredith et al. 2020). Briefly, a multiplex PCR-based approach was used according to the modified ARTIC version 2 protocol with version 3 primer set, and amplicon libraries sequenced using MinION flow cells version 9.4.1 (Oxford Nanopore Technologies, Oxford, UK). Sequences were made publicly available as part of COG-UK (https://www.cogconsortium.uk/, last accessed February 15, 2022) via weekly uploads with linked metadata onto the MRC-CLIMB server (https://www.climb.ac.uk/, last accessed February 15, 2022).

Samples collected via the CUH HCW screening program were also prioritized for onsite nanopore sequencing, as previously described (Rivett et al. 2020). This program entailed asymptomatic screening of selected wards, symptomatic testing of self-presenting HCW, and testing of symptomatic contacts of positive HCW. After an HCW tested positive, members of the HCW screening team contacted the HCW and retrospectively collected data on symptom onset date, symptomatology, household contacts, their job role, and which wards they had worked in for the preceding 2 weeks. Most positive HCW could identify symptoms on retrospective questioning, even if they were identified in the asymptomatic screening arm; however, a small minority were genuinely asymptomatic and never went onto develop symptoms. HCW presenting acutely to medical services at CUH were not part of the HCW screening program, but were identified as HCW from their medical records as part of hospital surveillance.

### Identifying Hospital-Associated Outbreaks for Investigation

Patients tested at CUH were categorized on the basis of time between admission and first positive swab into different groups reflecting the likelihood that their infection was community or hospital acquired, as previously described (Meredith et al. 2020). The categories used were 1) Community onset, community associated (first positive sample <48 h from admission and no healthcare contact in the preceding 14 days); 2) community onset, suspected healthcare associated (first positive sample <48 h from admission with healthcare contact in the preceding 14 days); 3) hospital onset, indeterminate healthcare associated (first positive sample 48 h to 7 days postadmission); 4) hospital onset, suspected healthcare associated (first positive sample 8–14 days postadmission); 5) hospital onset, healthcare associated (first positive sample >14 days postadmission); and 6) HCW.

We focused on hospital onset infections, defined as all CUH patients in categories 3, 4, and 5 (hospital onset with indeterminate, suspected, or definite healthcare associated COVID-19 infections) and 6 (HCW). The main wards, the HCW had worked in for the 2 weeks prior to testing positive and the ward where each patient had first tested positive were used to identify ward clusters of hospital-associated infections. Wards X and Y were among the five largest outbreaks of hospital onset COVID-19 from the “first wave” and used for retrospective analysis.

Ward Z was examined prospectively during the “second wave”—the infection control team highlighted this ward because it was a COVID-19 “red” ward (for confirmed COVID-19 patients) and several HCW working on the ward tested positive for SARS-CoV-2, despite the UK being under its second national lockdown (November 5 to December 2). The key question was whether the HCW could have been infected by the patients on the ward. All COVID-19 patients who had passed through Ward Z within 2 weeks of the HCW testing positive, and with sequence data available, and within three SNPs of either of the two HCW, were included for the *A2B-COVID* analysis.

### Prospective Clinical Application of *A2B-COVID* during the “Second Wave”

In the prospective analysis of clinical data during the “second wave,” the method was used by an Academic Clinical Fellow (T.F.) who was a member of the clinical team investigating the outbreaks. He was supervised by another Academic Clinical Fellow (W.L.H.) who had helped to develop the method. T.F. collected and curated the patient movement data, symptom onset data, and linked sequence metadata for the patients and HCW selected for further investigation from ward Z. Patient electronic medical records were reviewed for further evidence of direct contact between HCW and patients that A2B-COVID identified as having consistent transmission links (by W.L.H. and T.F.). In this way, the genomic data helped to focus which patients and HCWs should be prioritized for more indepth data collection efforts, during a period when the infection control team was under intense pressure. The results of the A2B-COVID analysis for Ward Z were presented by T.F. and T.G. at the weekly hospital COVID-19 review meetings and at a larger infection control meeting in early January 2021.

### Statistical Methods

The A2B-COVID method has been incorporated into a recently described approach for the construction of transmission networks among linked cases of SARS-CoV-2 infection (Illingworth et al. 2021). Where that approach is designed for command-line usage and can take considerable time (potentially several hours) to complete an analysis, the method described here has been implemented within an R package, available from http://github.com/chjackson/a2bcovid (last accessed February 15, 2022). A web interface to the package may be found at http://shiny.mrc-bsu.cam.ac.uk/apps/a2bcovid/ (last accessed February 15, 2022).

### Model Overview

We here consider pairs of individuals, who for the purpose of notation, we describe as individuals A and B. Given data on when the individuals became symptomatic for SARS-CoV-2 infection, their locations, and their viral genome sequences, we generate a statistic to test whether the data are consistent with the hypothesis that direct viral transmission occurred from A to B.

To outline this process, suppose that we have observed data (denoted y) from this pair of individuals. We first calculate a test statistic, describing the probability of observing y given transmission from A to B. Secondly, we compare this statistic to thresholds to identify whether these data are consistent with direct transmission from A to B, whether they are unlikely to have arisen from a direct transmission event, or whether this is a borderline case. Our thresholds are calculated from a sampling distribution governing the set of potential data (i.e., all data sets Y) that we could have observed from individuals who transmitted the virus one to another. Below we describe the calculation of the test statistic, then the calculation of the sampling distribution.

### Available Data

#### Notation

An overview of the notation used in the description of our model is shown in figure 4. The dates of symptom onset and the dates when viral sequence data were collected are denoted *S*_A_ and *S*_B_ and *D*_A_ and *D*_B_, respectively. Further data described the locations of the individuals A and B on each day, with the binary indicator *C*_A_(*L*, *T*) denoting whether individual A was present in location *L* on day *T*. The information describing the location of individuals may be uncertain, so we represent it by *w*_A_(*L*, *T*), the probability that individual A was present in location *L* on day *T*. Analogously to this, the binary indicator *C*_AB_(T) denotes whether or not A and B were in contact on day *T*. Uncertainty in this indicator is represented by the probability *w*_AB_(*T*) that A and B were present in the same location on this day. In describing genomic data, *H*_A_ and *H*_B_ describe Hamming distances between the viral sequences collected from A and B and their mutual consensus. The CT scores of the viral samples are denoted *V*_A_ and *V*_B_.

#### Symptom Onset Data

Due to extensive monitoring of individuals in hospital, we often had information on the dates of symptom onset for individuals. When these were unknown, symptom onset dates were estimated using corresponding positive test dates. An offset gamma distribution was fitted to model the difference between symptom onset and positive test dates from 86 health care workers and 393 patients from Cambridge University Hospitals (supplementary fig. S7 and table S1, Supplementary Material online). Where only a positive test date was known for an individual, the mean of this distribution was used to impute a symptom onset date. We write Ŝ_A_ to denote an estimate for S_A_. Where positive test dates are used in place of symptom onset dates, greater care is required in the interpretation of results from our method.

#### Location Data

Details of the locations of patients and health care workers were collected, describing which wards individuals were on each day. In our measurement of location data, we set *w*_A_(*L*, *T*) = 1 if an individual was known to be in location *L* for any part of day *T*. In order to account for the increased mobility of health care workers, night shifts which span more than 1 day, and uncertainties such as the potential for fomite transmission, we amended data collected for health care workers: If for a HCW we had that *w*_A_(*L*, *T*) = 1 for some *L* and *T* we set *w*_A_(*L*, *T*− 1) and *w*_A_(*L*, *T* + 1) to be equal to a minimum value of 0.5.

Where location data were missing it was necessary to specify values *w*_A_(*L*, *T*). In our study, data described cases from a specific part of the hospital, usually a single ward; this location was denoted L*. Where location data were missing for a patient, we set *w*_A_(L*, *T*) = 1 for all *T*, assuming that a patient was always on the most common ward. Where location data were missing for health care workers, we set *w*_A_(L*, *T*) = 4/7 for all *T*, reflecting shift patterns among workers. We note that in other circumstances (e.g., a data set spanning an entire hospital), an alternative prior for the location of individuals could be more appropriate.

Contact information was derived from the location data. For any two individuals we note that there could be multiple locations in which they could be colocated on a single day. We combined probabilities of contact across potential locations, calculating

$$w_{\mathrm{AB}}\left(t\right)=1-\prod_{L}\left({1-w}_{A}\left(L,t\right)w_{B}\left(L,t\right)\right).$$

#### Viral Genome Sequence Data

Consensus genome sequences were calculated from viral sequence data. Sequences were subjected to two levels of quality control. The first considered the coverage of the genome. An unambiguous nucleotide is here defined as an instance in which sequencing describes an A, C, G, or T. We applied the criterion that sequences had to unambiguously describe nucleotides at 80% or more of the sites in the genome.

Secondly, we considered sites in the genome that were polymorphic. These sites are more likely to be informative with regard to the number of genetic differences between two sequences; a genome with high overall coverage but ambiguity at multiple of these positions would in practice be quite uninformative. Having identified polymorphic sites, we required sequences to have no more than one ambiguous nucleotide at these positions.

In some cases, multiple viral samples were collected from the same individual. Viral genomes collected from the same individual were usually extremely similar to one another (supplementary fig. S1, Supplementary Material online). In such a case, we identified the earliest sequence with sufficient coverage of the viral genome, using this sequence for analysis. Where positions in this genome were ambiguous, and where other sequences from the same individual had unambiguous nucleotides at these positions, the other sequences were used to construct a more complete consensus sequence for the individual.

Given viral sequences from the pair of individuals A and B we calculated Hamming distances from each sequence to a pairwise consensus sequence; we denote these distances as *H*_A_ and *H*_B_.

### Assessing Viral Transmission

We denote as *X*_T_ an indicator for the event that transmission took place at time *T*, and as *X* is an indicator for the event that transmission took place at all. To test the hypothesis of transmission, we calculated a test statistic defined by the probability *p*(*y*|*X*) of observing the data y under the null hypothesis that transmission occurred. We note that

(1) $$p(y|X)=\sum_{T}P(y{|X}_{T})P\left(X_{T}|X\right)$$

where *P*(*X_T_*|*X*) is the probability that transmission took place at time *T* given that transmission occurred. For simplicity, we write *P*(*T*) = *P*(*X_T_*|*X*).

We now let *Y* represent an example of potentially observable data from a pair of individuals. To test the null hypothesis of transmission, we first need to determine the sampling distribution of *Y* given transmission, which is used both to calculate the test statistic *p*(*y*|*X*) and its null distribution. *Y* consists of the symptom time *S*_B_, the Hamming distances *H*_A_ and *H*_B_, and the set of *C*_AB_(*T*) for all *T*, denoted *C*_AB_. The probability of observing *Y* given transmission is given by:

(2) $$p(Y|D,X,\theta )=\sum_{T}P(T|S_{A},\theta )P\left(S_{B}|\theta ,X_{T}\right){P(C}_{\mathrm{AB}}|X_{T})P\left(H_{A},H_{B}|\;\theta ,{D,E,X}_{T}\right)$$

where *D* = {*D*_A_, *D*_B_}, *E* is the error in sequencing, and θ represents a set of known parameters. We note that we condition on *S*_A_; an alternative approach would be to write the equation in terms of *S*_B__–_*S*_A_. We consider the parts of this equation in turn.

### Assessing Viral Transmission: Symptom and Location Data

In equation (2), *P*(*T*|*S*_A_,θ) describes the probability that transmission is at time *T*, where time is measured relative to *S*_A_, the time of onset of symptoms in A. This term describes the infectivity profile of the virus, that is, the time from symptom onset to transmission. We follow previously published work which has characterized this as an offset gamma distribution (He et al. 2020; Li et al. 2020; Ashcroft et al. 2020).

The term *P*(*S*_B_|θ,*X_T_*) describes the probability that B becomes symptomatic at time *S*_B_, given that transmission occurs at time *T*; this has been characterized in the same literature as a lognormal distribution. We therefore write:

(3) $$P(T|S_{A},\alpha ,\beta ,s)=\frac{e^{-(T-S_{A}+s)/\beta }{\left(T-S_{A}+s\right)}^{\alpha -1}\beta^{-\alpha }}{\Gamma \left(\alpha \right)},$$

where *s* is the offset and α = 97.1875, β = 0.2689, and *s* = 25.625, and

(4) $$P\left(S_{B}|\mu ,\sigma ,X_{T}\right)=\frac{e^{-{(\left(\log\left(S_{B}-T\right)-\mu \right)}^{2}/2\sigma^{2})}}{\left(S_{B}-T\right)\sigma \sqrt{2\pi }},$$

where μ = 1.434 and σ = 0.6612.

Although all calculations in this manuscript were performed on data describing infection with the original pandemic strain, the Delta variant has a shorter incubation period and time to peak viral load (Li et al. 2022; Ong et al. 2021). To account for this, we implemented an option to use parameters derived in a recent paper for the Delta variant; namely with the values α_Δ_ = 38.4805, β_Δ_=0.468049, and s_Δ_=20; μ_Δ_=1.39599, and σ_Δ_=0.41354 (Kemp et al. 2021). Distributions generated by these parameter sets are shown in supplementary figure S8, Supplementary Material online.

Each of these expressions treat *T* as a continuous variable; we used an approximation to discretize the formula to a resolution of single days, obtaining

(5) $$P(T|S_{A},\theta )P\left(S_{B}|\theta ,X_{T}\right)=\left[\int_{T-S_{A}-0.5}^{T-S_{A}+0.5}\frac{e^{-(x+s)/\beta }{\left(x+s\right)}^{\alpha -1}\beta^{-\alpha }}{\Gamma \left(\alpha \right)}dx\right]\left[\int_{S_{B}-T-0.5}^{S_{B}-T+0.5}\frac{e^{-{\left(\log\left(x\right)-\mu \right)}^{2}/2\sigma^{2}}}{x\sigma \sqrt{2\pi }}dx\right].$$

We next consider the term *P*(*C*_AB_|*X_T_*), which describes the probability of a pattern of colocation between A and B given that transmission occurred on day *T*. We first note that colocation is necessary for transmission on day *T*, giving *P*(*C*_AB_(*T*) = 1|*X_T_*) = 1 and *P*(*C*_AB_(*T*) = 0|*X_T_*) = 0. Secondly, we assume that knowledge of whether A and B were colocated at times *other than T* does not give any information relevant to the hypothesis of transmission at *T*. Therefore, we define *P*(*C*_AB_(*t*) = 1|*X_T_*) = *P*(*C*_AB_(*t*) = 0|*X_T_*) = 0.5 for each *T* and *t* ≠*T*, which ensures that any observed pattern of colocation at the same number of times other than *T* will have identical probability given *X_T_*, hence will lead to the same contribution to *p*(*y*|*X_T_*). The number of potential transmission times under consideration (denoted |*C*|) is the same for all pairs A–B in our data, hence the probability for any pattern of colocation at times *t* ≠*T* is identically 0.5^|*C*|−1^ for each *T*. Hence the location data only influence the test statistic *p*(*y*|*X*) through ruling out transmission at times where A and B were not in the same place.

Next consider the contribution of the *observed* colocation data to the test statistic. Recall that the observed colocation status is uncertain for many pairs of individuals A and B in our data. Our knowledge is described by the values *w*_AB_(*t*) for each pair A and B, and for each time *t*, derived either from explicit data describing the colocation of A and B, or by judgments and assumptions made in the absence of data. In supplementary text S1, Supplementary Material online, we generalize our calculation above to show that in this case

(6) $$p\left(C_{\mathrm{AB}}|X_{T}\right)=0.5^{\left|C\right|-1}w_{\mathrm{AB}}\left(T\right).$$

### Assessing Viral Transmission: Viral Sequence Data

Finally, we consider the term *P*(*H*_A_, *H*_B_|θ,*D*, *X_T_*), which is derived from the viral genome sequence data. Following an approach based on phylogenetic reconstruction, we generated an outgroup sequence as the consensus of all of the viral genomes in our data. For a given pair of sequences from individuals A and B, we then calculated a local consensus, defined as the nucleotide shared by the two sequences where the sequences agreed, and the nucleotide in the outgroup where the sequences differed. The values *H*_A_ and *H*_B_ were then calculated as the Hamming distances from each of the two sequences to the local consensus. These distances describe the number of substitutions gained by the viral population in each individual since the time of the most recent common ancestor.

We used a Poisson model to compare the number of observed substitutions in each sequence with an expected rate of viral evolution. Our model includes a term accounting for errors in the viral consensus sequences. Adopting an infinite sites model, we assume that in the short periods of time considered, changes to the viral consensus sequence can occur, but cannot revert. In the notation of figure 4, we then note that if *D*_A_ is before *T*, any variants observed in sequence data from A but not in the data from B can only arise from error, with no possibility for a variant reverting between *D*_A_ and *D*_B_. Alternatively, if *D*_A_ is after *T*, such variants have the potential to occur in the time between *D*_A_ and *T*.

By a similar logic, variants observed in data from B but not from A can arise either from error, or as a result of evolution going back to the most recent common ancestor, found at the earlier of the times *T* and *D*_A_. We therefore have the result:

(7) $$P\left(H_{A},H_{B}\;|\;\theta ,{D,E,X}_{T}\right)=\left(\frac{{\left(E/2+\gamma_{G}P_{A}\right)}^{H_{A}}e^{-\left(E/2+\gamma_{G}P_{A}\right)}}{H_{A}!}\right)\left(\frac{{\left(E/2+\gamma_{G}\left({D_{B}-Q}_{A}\right)\right)}^{H_{B}}e^{-\left(E/2+\gamma_{G}\left({D_{B}-Q}_{A}\right)\right)}}{H_{B}!}\right)$$

where *P*_A_ = max{0, *D*_A_ − *T*} and *Q*_A_ = min{*D*_A_, *T*}. The rate of evolution γ_G_ describes the expected number of substitutions per genome per day, whereas the parameter *E* is the mean number of errors in the Hamming distance between two viral sequences, estimated as described below.

### Estimating Noise in Genome Sequence Data

In order to estimate the extent of measurement error in a consensus viral genome, we examined cases among data collected at Cambridge University Hospitals (CUH) for which more than one viral sample was sequenced. We identified 136 such patients, with between two and nine samples collected from each individual and 336 samples in total. Each sample gave rise to a consensus sequence; we filtered the data to remove sequences with <90% coverage of the genome. Intervals between pairs of samples varied from 0 to 39 days. For each pair of samples i and j, collected from the same individual, we recorded *H*_ij_, the Hamming distance between them, Δ*T*_ij_, the absolute difference in time between the dates on which the samples were collected, measured in days, and the viral load of each sample, as represented by the CT scores *V*_i_ and *V*_j_.

Following in principle a previous approach to estimating noise and rates of evolution (Lumby et al. 2020), we then fitted a Poisson model to the data, deriving for each pair the log likelihood

(8) $$\log\;L^{D}\left({\varepsilon ,\lambda ,\gamma \;|\;H}_{\mathrm{ij}},{\Delta T}_{\mathrm{ij}},V_{i},V_{j}\right)=\log\left(\frac{{\left(\frac{\varepsilon }{2}\left(V_{i}+V_{j}\right)+\lambda +\gamma \Delta T_{\mathrm{ij}}\right)}^{H_{\mathrm{ij}}}e^{-\left(\frac{\varepsilon }{2}\left(V_{i}+V_{j}\right)+\lambda +\gamma \Delta T_{\mathrm{ij}}\right)}}{H_{\mathrm{ij}}!}\right)$$

and estimating the parameters ε, λ, and γ so as to maximize the sum of the log likelihoods across all pairs of sequences; we inferred the parameters $\hat{\epsilon }$ = 0.0200, $\hat{\lambda }\;$=−0.0693, and $\hat{\gamma \;}$= 0.0453. Here, the value $\hat{E}\left(V_{i},V_{j}\right)=\hat{\lambda }\;+\hat{\epsilon }(V_{i\;}+V_{j})$ provides a very simple estimate of the extent of measurement error in a Hamming distance, expressed in terms of the mean CT score of the two samples. For the purposes of our model, this function was evaluated at the mean CT score of 24.091. This provided an estimate for the pairwise difference arising through measurement error, $\hat{E}$, of 0.414 nucleotides, equivalent to 0.207 nucleotide errors per genome sequence. The estimate $\hat{\gamma }$ describes the mean rate of within-host evolution calculated across the within-host sample. It is expressed as a number of substitutions per genome per day, and is equivalent to a rate of 6.0 ×10^−4^ substitutions per locus per year, close to the value of 8×10^−4^ that has been calculated from global sequence data (Hadfield et al. 2018). In so far as we require an estimated rate of evolution spanning both within-host and between-host evolution, we used in our model a rate $\hat{\gamma_{G}}$ of 0.0655 nucleotides per day, equivalent to this latter, globally estimated, rate of evolution.

To examine the effect of CT score upon our inference, a repeat calculation was performed in which these data were ignored; while our model of CT score is somewhat crude, omitting it gave a worse fit to the data under the Bayesian Information Criterion (Schwarz 1978; Hadfield et al. 2018) (supplementary table S2, Supplementary Material online).

In a case where no sequence data were observed for an individual, we excluded that individual from our calculation. An option within our method allows for calculations to be performed between individuals where no sequence data were collected; under this option we set *P*(*H*_A_, *H*_B_|θ, *D*, *E*, *X_T_*) = 1 for all A and B.

### Assessing Viral Transmission: Hypothesis Testing

Having derived the expression (2) for *P*(*Y*|*D*, *X*), we now derive the probability *P*(*y*|*D*, *X*) of the specific observed (as opposed to observable) data *y*. The data *y* consist of the symptom time *S*_B_, if it is known, the Hamming distances *H*_A_ and *H*_B_, the set of those *C*_AB_(*T*) that are known, and the information about potential locations and colocations in cases where the *C*_AB_(*T*) are unknown, which are encapsulated in *w*_AB_(*T*). We obtain *p*(*y*|*D*, *X*) from *P*(*Y*|*D*, *X*), setting *Y* to equal the data *y* that are observed, and then integrating *P*(*Y*|*D*, *X*) over the potential values for any missing data.

Including the expression for *P*(*C*_AB_|*X_T_*) derived above gives us the result

(9) $$p(y|D,X)=\sum_{T}P(T|\hat{S_{A}},\theta )P\left(\hat{S_{B}}|\theta ,X_{T}\right){0.5^{|C|-1}w}_{\mathrm{AB}}(T)P\left(H_{A},H_{B}|\;\theta ,{D,X}_{T}\right)$$

where θ = {α, β, s, μ, σ, Ê, γ_G_}.

In order to test the hypothesis of whether the data *y* are consistent with transmission, we compare the value *p*(*y*|*D*, *X*) to the set of possible values *p*(*Y*|*D*, *X*) for potential data *Y*, and identify thresholds *p*(*y*|*D*, *X*) = ψ at which we can reject this hypothesis.

We defined the space Ω as the set of all possible *Y*, and constructed an ordering of all of the *Y* ∈ Ω, so that *p*(*Y*_i_|*D*, *X*) ≤ *p*(*Y*_j_|*D*, *X*) if *i* ≤ *j*. Next, we identified threshold sets, *Y*_T1_ and *Y*_T2_, defined so that *T*_1_ and *T*_2_ are the smallest integers satisfying

$\sum_{i=1}^{i=T_{1}}p\left(Y_{i}|D,X\right)\;\geq \;0.95\sum_{Y\in \Omega }p\left(Y|D,X\right)$ and $\sum_{i=1}^{i=T_{2}}p\left(Y_{i}|D,X\right)\;\geq \;0.99\sum_{Y\in \Omega }p\left(Y|D,X\right).$

In this way, we defined thresholds

$$p_{95\;}\left(D\right)=p\left(Y_{T_{1}}|D,X\right)$$

and

$$p_{99\;}\left(D\right)=p\left(Y_{T_{2}}|D,X\right).$$

The observed data *y* were then deemed “consistent” with transmission if *p*(*y*|*D*, *X*) ≥ *p*_95_(*D*), “borderline” if *p*_95_(*D*) > *p*(*y*|*D*, *X*) ≥ *p*_99_(*D*), and “unlikely” if *p*_99_(*D*) > *p*(*y*|*D*, *X*). Details of the calculation of threshold values are given in supplementary text S2, Supplementary Material online.

We note that where *C* is defined to have a consistent length for all pairs A and B, it contributes a constant term 2^|*C*|−1^ to *p*(*y*|*D*, *X*) and to each value *p*(*Y*|*D*, *X*), so that it can be neglected in the comparison of outputs to thresholds.

We note that our definition of data being “consistent” with transmission is somewhat arbitrary, identifying, as reflected in our simulation results, 95% of direct transmission events alongside a proportion of cases in which individuals were not related via direct transmission. Our approach provides a heuristic assessment of data to assist the targeting of further epidemiological investigation.

### Simulated Transmission Events

We generated examples of direct and indirect SARS-CoV-2 transmission events based upon the infectivity profile and time to symptom onset of the virus. Details of simulations, and the generation of conditional offset gamma distributions which enable these to be performed, are given in supplementary text S3, Supplementary Material online.

### Use of Sequence Similarity Cutoff

The Mathematica software package (v12.3.1.0) was used to calculate sequence distances between aligned sequences and to produce supplementary figure S5, Supplementary Material online. In these calculations ambiguous nucleotides were ignored.

## Ethical Statement

This study was conducted as part of surveillance for COVID-19 infections under the auspices of Section 251 of the NHS Act 2006. It therefore did not require individual patient consent or ethical approval. The COG-UK study protocol was approved by the Public Health England Research Ethics Governance Group (reference: R&D NR0195).

## Supplementary Material

Supplementary data are available at *Molecular Biology and Evolution* online.

## Author Contributions

C.I., W.L.H., B.W., M.R., A.P., T.G., D.d.A., and M.E.T. conceptualized the study; W.L.H., A.P., L.M., C.J.H., M.H., A.J., M.R., B.W., L.C., S.C., A.Y., G.H., F.A.K., T.F., M.P., I.Ge., Y.C., M.C., S.P., D.S., L.R., N.J., S.S., S.F., T.D., K.G., C.W., E.G.K., N.M.B., M.P.W., S.B., and M.E.T. were responsible for data curation and investigation; C.I., W.L.H., C.J., A.P., B.W., M.R., and M.E.T. conducted the formal analysis; S.J.P., I.G., S.B., M.P.W., M.E.T., and E.G.K. acquired funding; C.I., W.L.H., and C.J. designed the methodology; T.G., I.G., D.d.A., and M.E.T. administered the project; M.C., S.P., N.M.B., M.P.W., S.B., and I.G. provided resources; C.I., W.L.H., and C.J. contributed software; S.J.P., I.G., T.G., D.d.A., and M.E.T. were responsible for supervision; C.I., W.L.H., C.J., and T.G. carried out validation; C.I., W.L.H., and C.J. designed visualizations; C.I., W.L.H., and M.E.T. wrote and prepared the original draft. All authors wrote, reviewed, and edited the manuscript.

## Supplementary Material

msac025_Supplementary_Data
