# Supplementary material for: A2B-COVID: A Tool for Rapidly Evaluating Potential SARS-CoV-2 Transmission Events
Source: Mol Biol Evol. 2022 Feb 2;39(3):msac025. doi: 10.1093/molbev/msac025 (PMC8892943; doi:10.1093/molbev/msac025)
Supplement: msac025_Supplementary_Data [file msac025_supplementary_data.pdf]

Supplementary Information

Supplementary Figures

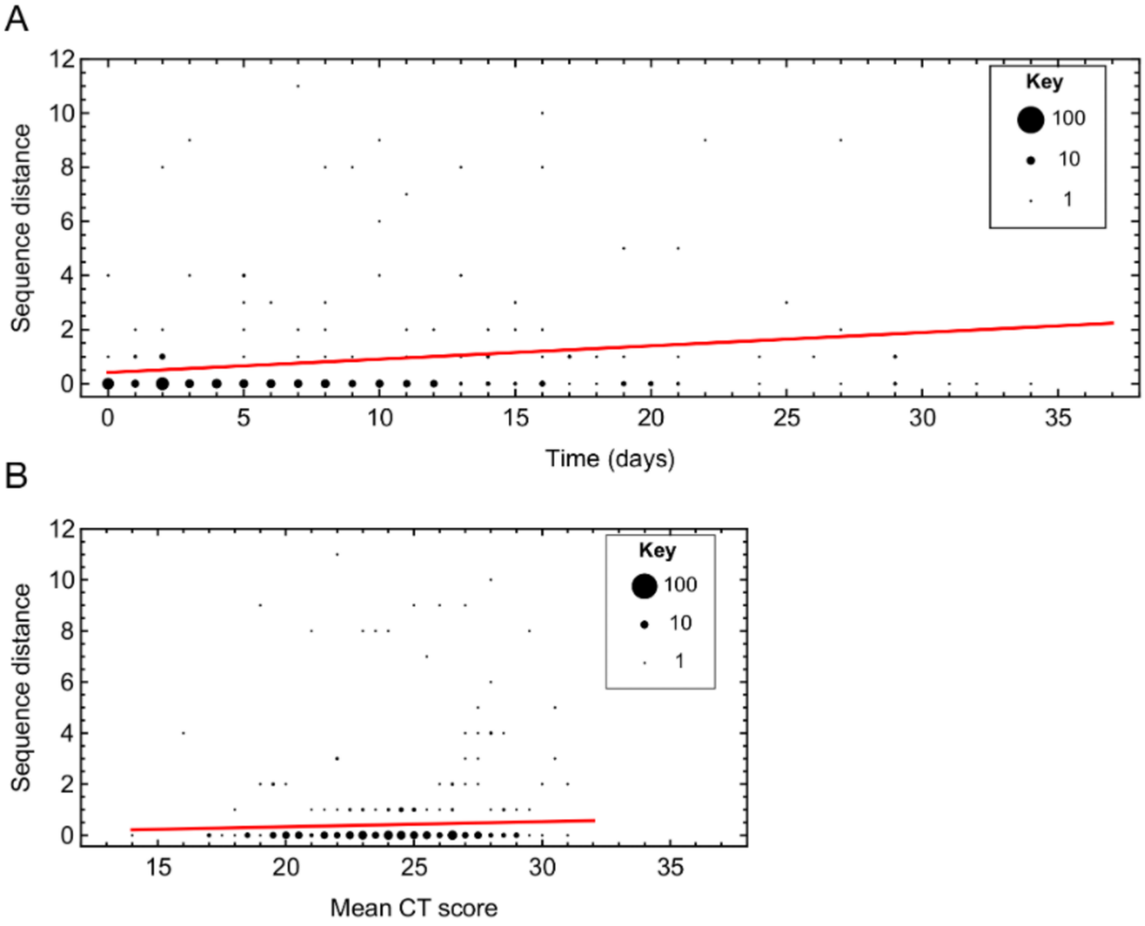

Supplementary Figure S1: Analysis of Hamming distances between pairs of genome sequences collected from viral samples in the same host. Figures show projections through a multi-linear model fit to the data using a Poisson likelihood. **A.** Relationship between the Hamming distance and time between samples. The line shows the fit to the data at the mean CT score. The size of a dot is proportional to the number of pairs with given parameters. **B.** Relationship between the Hamming distance and mean CT score of the two samples. The line shows the fit to the data calculated at zero time between samples.

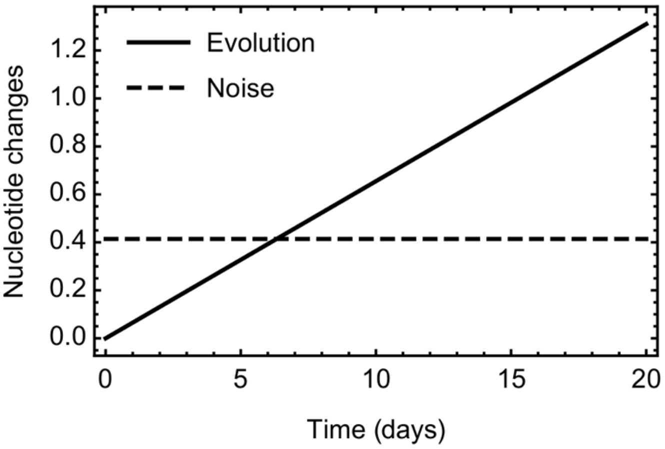

Supplementary Figure S2: Comparison between the expected rate of SARS-CoV-2 evolution within our model, and the expected difference between two sequences caused by noise. The expected time between symptoms being reported from individuals in a transmission pair is 5.7 days, in which time the expected number of substitutions arising via evolution is 0.373. The expected number of differences between two genome sequences resulting from error was estimated as 0.414.

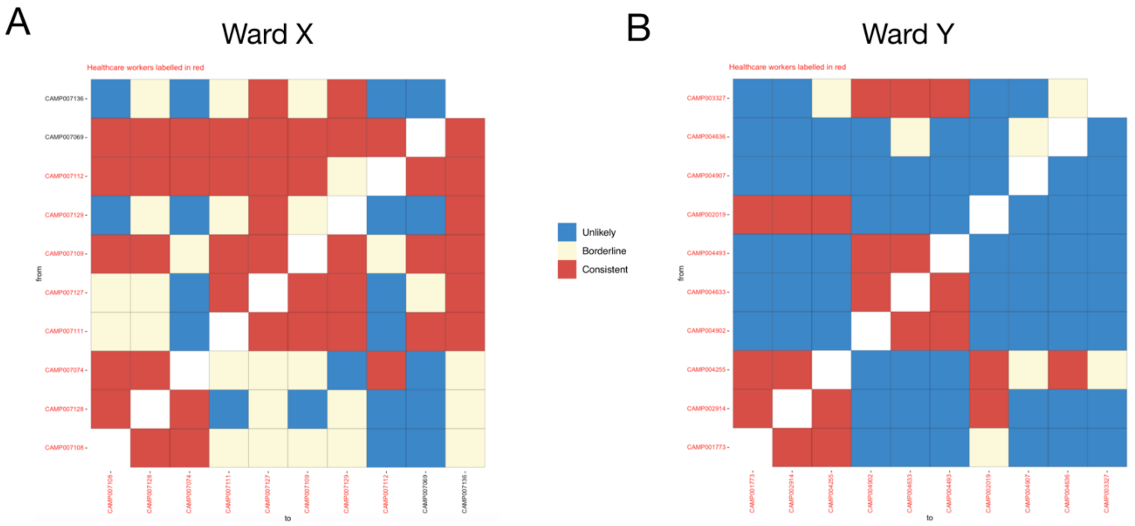

Supplementary Figure S3: Analysis of partial data collected from wards X and Y, omitting location data. A. Output from the A2B-COVID package given data from ward X, omitting location data for individuals. The plot shows potential links between infections. Identifiers of individuals are coloured in either black (patients) or red (HCWs). Squares in the grid indicate that transmission from one individual to another is consistent with our model (red), borderline (yellow) or unlikely (blue). **B.** Output from the A2B-COVID package given data from ward Y, omitting location data for individuals.

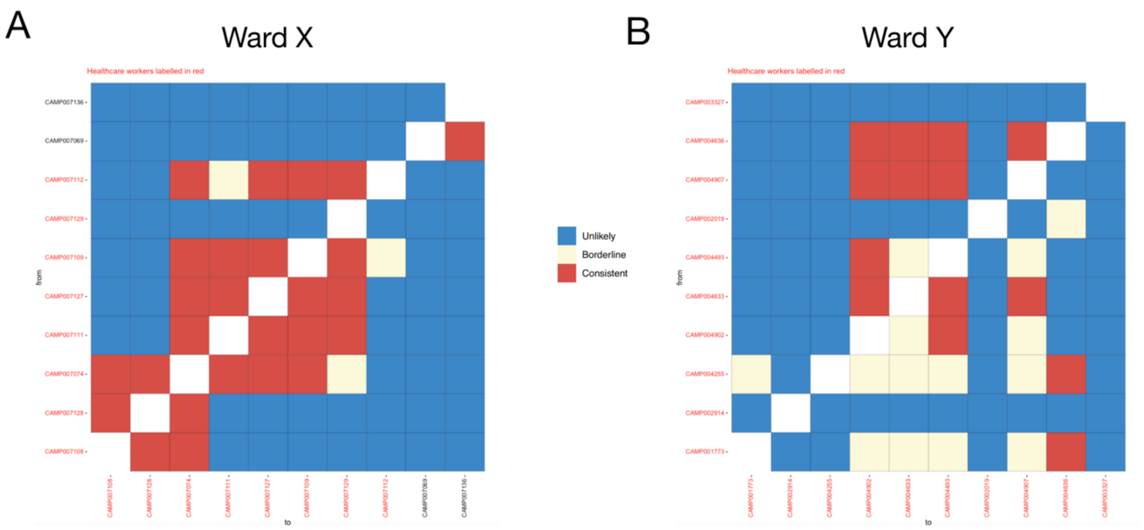

Supplementary Figure S4: Analysis of partial data collected from wards X and Y, omitting sequencing data. A. Output from the A2B-COVID package given data from ward X, omitting sequence data for individuals. The plot shows potential links between infections. Identifiers of individuals are coloured in either black (patients) or red (HCWs). Squares in the grid indicate that transmission from one individual to another is consistent with our model (red), borderline (yellow) or unlikely (blue). **B.** Output from the A2B-COVID package given data from ward Y, omitting sequence data for individuals.

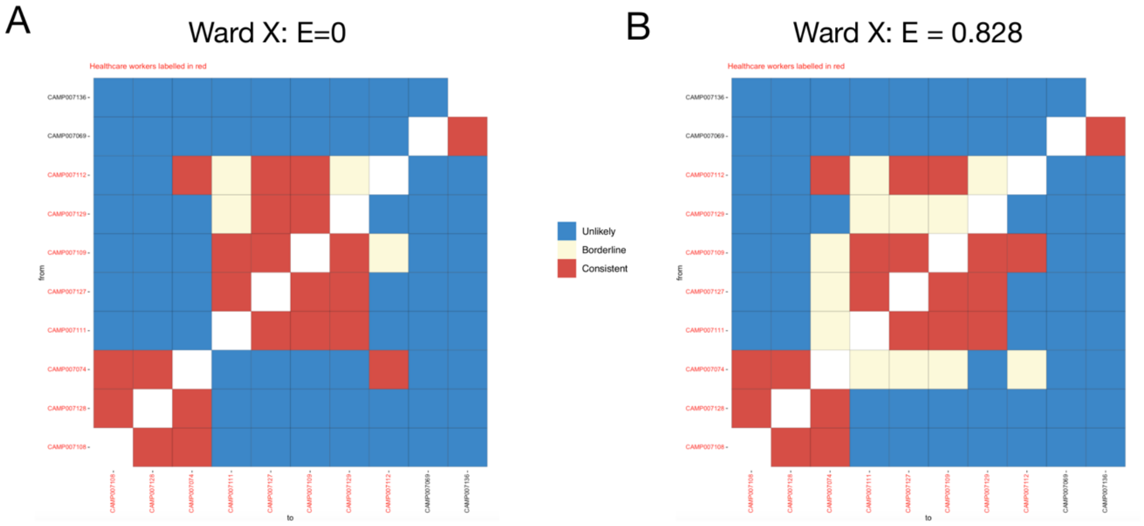

Supplementary Figure S5: Results generated using a simple sequence cutoff. An evaluation was performed of sequences from the individuals in each of our wards, identifying cases for which genome sequences were separated by no more than two unambiguous nucleotides, but with no other criteria for identifying consistency with a transmission model. Here cases are marked as 'consistent' with transmission in red, or as 'unlikely' to be associated in blue. We note that A2B-COVID does not perform this analysis; the Mathematica software (v12.3.1.0) package was used to calculate Hamming distances between sequences.

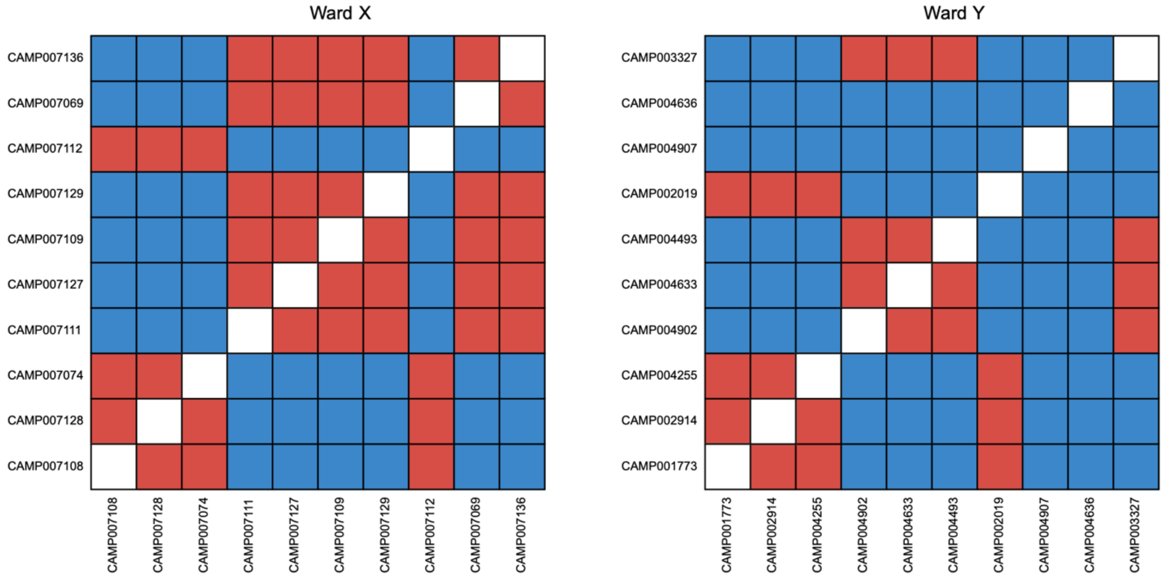

Supplementary Figure S6: Sensitivity of our results to changes in the extent of error in sequencing. Changing the parameter which determines the extent of error in a genome sequence led to small changes in the inferred model output. **A.** Inferences of potential transmission events for ward X given a noise parameter of zero. **B.** Inferences of potential transmission events for ward X given a noise parameter double that inferred from our data.

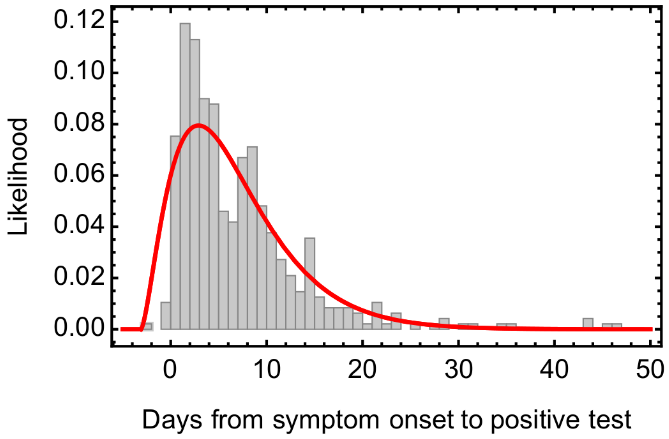

Supplementary Figure S7: Raw data (bars) and inferred model (red line) describing the distribution of the time between the onset of symptoms and receiving a positive test. This model was used to impute equivalent symptom onset dates for individuals who were asymptomatic or for whom no data on symptom onset date were available.

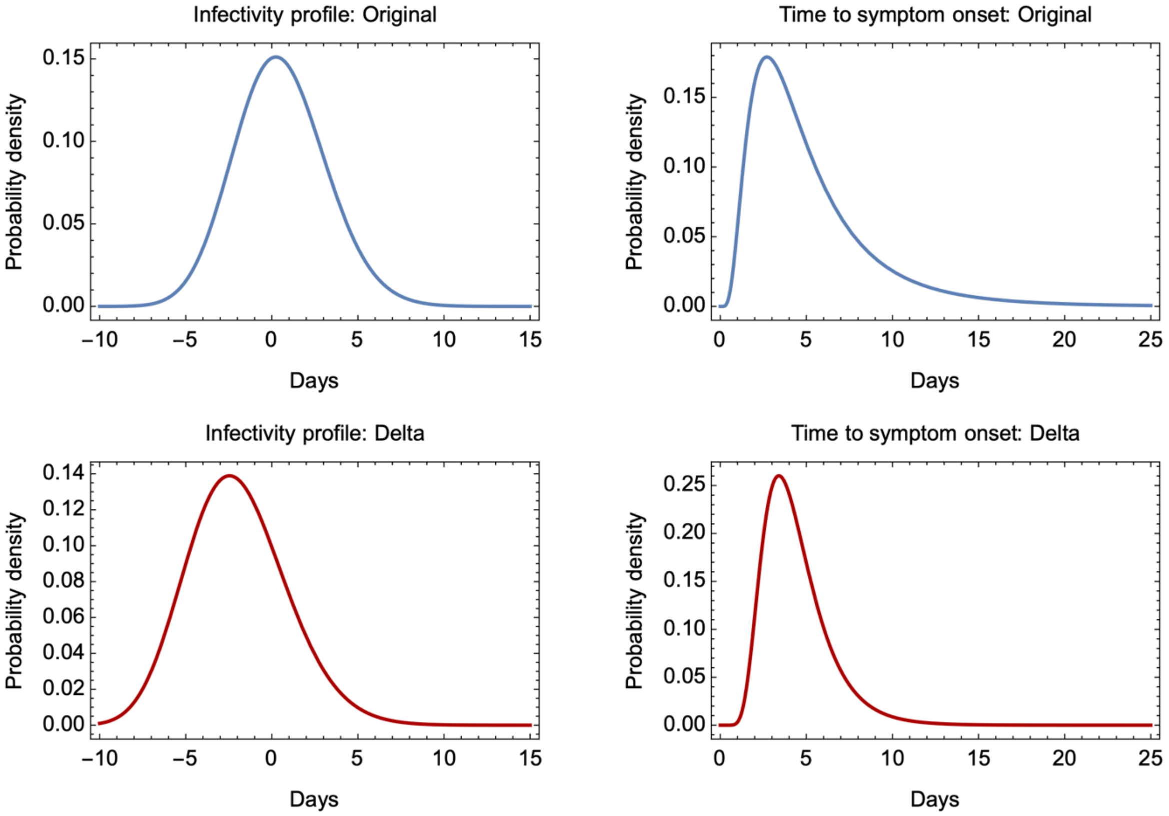

Supplementary Figure S8: Distributions describing the infectivity profile and the time to symptom onset within our standard model, and within a second model parameterised to fit the Delta variant of SARS-CoV-2[37].

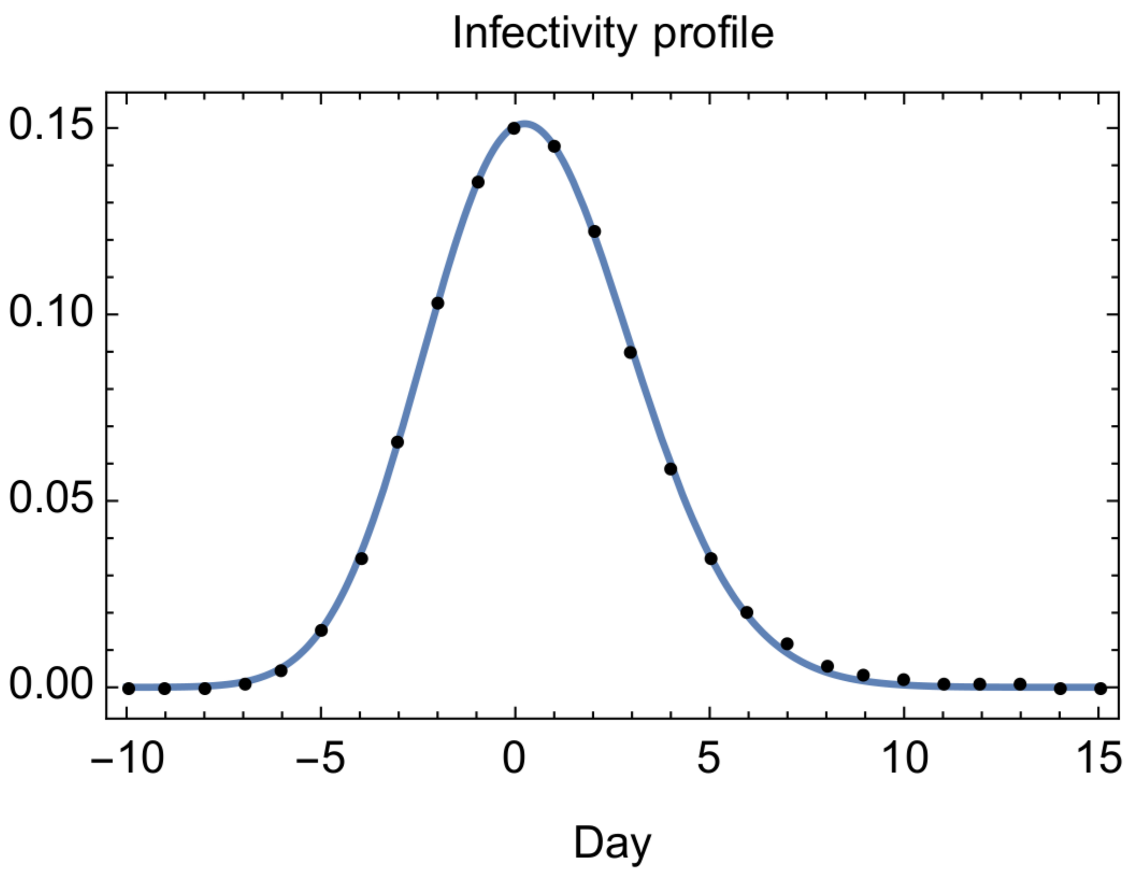

Supplementary Figure S9: Approximation of the infectivity profile used in simulations. The blue line shows the basic infectivity profile, described by an offset gamma distribution. The black dots show an approximation to this distribution, derived for the purposes of simulating data. The approximation consists of a sum of offset gamma distributions, describing in turn the infectivity profile conditional on an individual becoming symptomatic a fixed number of days after being infected. Within the simulation regime, the day of symptom onset is used to condition the infectivity profile so that an individual cannot infect someone else before they are themselves infected.

Supplementary Tables

Table S1: Parameters for the offset gamma distribution fitted to data describing intervals between times of reporting symptoms and positive test results. Inferred values were generated using maximum likelihood; the range describes a window of size two likelihood units from the maximum.

**Model**	**Inferred distribution**
**Parameter**	**Value (Range)**
**α**	2.593 (2.269, 2.976)
**β**	3.776 (3.292, 4.353)
**Offset (o)**	3.112 (3.011, 3.431)

Table S2: Error models fitted to Hamming distance data. A model incorporating dependence upon the time between samples and upon viral load gave the best fit to the data. The error parameter used was calculated at zero time between samples and at the mean viral load.

**Model**	**Parameters**			**BIC**
	**Constant (λ)**	**Time dependence (γ)**	**CT dependence (α)**	
Constant error	0.842			887.3
Time-dependent	0.385	0.0525		858.5
Time and CT dependent	-0.0692	0.0492	0.0200	857.8

Table S3: Inferred parameters in a conditional SARS-CoV-2 infectivity profile, used to model transmission events. The value x describes the number of days between an individual being infected and becoming symptomatic, while the alpha and beta parameters describe shifted gamma distributions, representing conditional infectivity profiles for each x.

x	α_x	β_x
1	6.29887	0.60263
2	3.6005	1.07799
3	2.98496	1.46698
4	3.08435	1.37114
5	4.66787	1.16352
6	4.9009	1.01113
7	7.3627	0.610795
8	14.271	0.657678
9	11.7093	0.441194
10	19.7355	0.569267
11	22.3441	0.544431
12	25.9136	0.506002
13	27.8563	0.501716
14	38.5445	0.399918
15	37.8481	0.423175
16	43.7543	0.391412

17	52.9138	0.347598
18	54.2553	0.351672
19	61.0491	0.330609
20	64.9268	0.325558
21	71.2873	0.309859
22	77.0822	0.299738
23	84.9344	0.28421
24	89.8652	0.204254
25	96.4369	0.27019
26	100.161	0.205282
27	123.77	0.228947
28	131.131	0.223556
29	139.907	0.216391
30	137.009	0.227041
31	169.678	0.191274
32	154.982	0.213549
33	154.663	0.21955
34	186.97	0.188751
35	199.226	0.1824

36	192.191	0.192526
37	204.034	0.186639
38	215.246	0.181583
39	222.968	0.17925
40	239.513	0.171731
41	256.03	0.164586
42	260.162	0.165742
43	271.968	0.162102
44	303.796	0.148823
45	300.115	0.153521
46	303.952	0.135088
47	320.938	0.149763
48	333.349	0.14722
49	391.487	0.128674
50	366.126	0.139737
51	373.742	0.139248
52	381.284	0.138968
53	401.472	0.134543
54	397.146	0.13808

55	425.354	0.131625
56	438.84	0.129785
57	454.595	0.127467
58	464.054	0.126995
59	478.7	0.125191
60	491.683	0.12383
61	541.534	0.114652
62	524.632	0.119918
63	533.974	0.119573
64	578.311	0.112372
65	569.142	0.115745
66	571.488	0.116809
67	584.427	0.115898
68	599.162	0.114811
69	609.809	0.114222
70	620.2	0.113911
71	634.211	0.112915
72	644.58	0.112615
73	727.031	0.101639

74	691.864	0.107814
75	679.966	0.110856
76	746.175	0.102855
77	746.113	0.104125
78	725.261	0.10808
79	722.592	0.109749

Supplementary Text

Supplementary Text S1: Further methodological details

In the main text we stated that:

$P(C_{AB} \mid X_T) = 0.5^{|C|-1} w_{AB}(T)$

To derive this result we note that, if it is observed that $C_{AB}(T)=0$, transmission cannot occur at time T, so that $P(C_{AB}|X_T)=0$. If it is observed that $C_{AB}(T)=1$, we next consider the element $C_{AB}(t)$ of C_{AB} for a time $t \neq T$. If $C_{AB}(t)$ is observed, we apply our approach to contact patterns, assuming that $P(C_{AB}(t)=1|X_T) = P(C_{AB}(t)=0|X_T) = 0.5$, such that the probability of this observation is 0.5. If $C_{AB}(t)$ is not observed, its probability is obtained by integration. We have that $P(C_{AB}(t) \mid X_T) = w_{AB}(t) * 0.5 + (1 - w_{AB}(t)) * 0.5 = 0.5$. Hence if $C_{AB}(T)=1$, the probability $P(C_{AB} \mid X_T)$ of the whole contact vector is equal to $0.5^{|C|-1}$. Finally, we consider the case in which $C_{AB}(T)$ is missing data. Integrating over the missing value, we have that

$P(C_{AB}(T) \mid X_T) = w_{AB}(T) * P(C_{AB} \mid X_T, C_{AB}(T)=1) + (1 - w_{AB}(T)) * P(C_{AB} \mid X_T, C_{AB}(T)=0) = w_{AB}(T)$

Applying again the reasoning above, this gives us the result $P(C_{AB} \mid X_T) = 0.5^{|C|-1} w_{AB}(T)$. As we defined $w_{AB}(T) = C_{AB}(T)$ when $C_{AB}(T)$ was observed, we thus have that $P(C_{AB} \mid X_T) = 0.5^{|C|-1} w_{AB}(T)$ in every case.

Supplementary Text S2: Calculation of threshold values.

To generate threshold values we calculated p(Y|D,X) across large numbers of sets of data Y, in which we assumed without loss of generality that S_A=0. Calculations were performed for all Y in which $S_B \in [-11, 87]$, and for all values H_A and H_B for which $H_A+H_B \in [0, 10]$ and $H_A \in [0, H_A+H_B]$; these ranges were chosen to return values of at least 10^{-6} from each component of p(Y|D,X). The ranges used give a good approximation to the total reasonable set of all Y. In our code statistics were calculated for $D_A \in [-10, 40]$, and $D_B \in [S_B-10, S_B+40]$; values outside of these ranges are unlikely.

In the integral, we note that there are a large number of possible vectors C_{AB} that indicate all times when a pair were in contact. We approximated the sum by generating 100 random vectors C_{AB} for each set of other parameters, and calculating the sum over these vectors, altering the value $0.5^{|C|-1}$ in $P(C_{AB}|X_T)$ so as to normalise the integral. Reflecting our approach to contact patterns, we generated the C_{AB} as random vectors of draws from a Bernoulli distribution with probability 0.5. Repeating this calculation with different sets of 100 vectors did not substantially change the thresholds obtained. Our code allows for the generation of alternative thresholds with different probabilities of an element of C_{AB} being equal to 1. We note that if this probability is higher, fewer datasets will be judged consistent with transmission.

Supplementary Text S3: Simulations

The Mathematica software package v12.3.1.0 was used to generate simulated data. An individual A was assumed to be infected on day 0. After this, A infected either the individual B, or a chain of n individuals before, the last of which infected B. Upon being infected, an individual became symptomatic a number of days afterwards, according to the symptom onset distribution used within A2B-COVID. The time at which an individual infected another was calculated using an offset gamma distribution, with parameters calculated conditionally upon the time of symptom onset, as described below. A sample was collected for sequencing a whole number of days between 2 and 10 days after symptom onset, this value being calculated from a uniform distribution. The number of substitutions in each genome sequence was calculated as a Poisson distribution, with parameter equal to the sum of two values. The first value was calculated as the rate of evolution used within A2B-COVID multiplied by the time from the divergence in the transmission tree between A and B, and the time at which a sample was collected. We note that divergence occurs at the time when A infects another individual. The second value represents noise, and was specified according to the value inferred from within-host data.

Simulated data was analyed using a cut-down version of the A2B-COVID code, called A2B-Core, implemented in C++, which facilitates rapid calculations across large numbers of pairs of individuals without the use of an R interface. The code for A2B-Core is included in the Github for A2B-COVID.

Conditional parameters for an offset-gamma distribution

We first note that generating simulated data requires some elaboration of the distributions used in our study. While the time to symptom onset has a mean of about 5.2 days, the time between symptom onset and infecting another individual, specified by the infectivity profile, has a range of possible outputs, starting at -25 days. If the two distributions are considered independently, it is therefore possible to generate a case in which individual A infected individual B at a time before individual A was infected with the virus.

To solve this problem we generated a conditional infectivity profile. Suppose that individual A became infected on day zero. Then the time S_A at which A became symptomatic is given by a distribution similar to that of equation 4.

$$P(S_A = x|\mu, \sigma, X_0) = \frac{e^{-\frac{(log(x)-\mu)^2}{2\sigma^2}}}{x\sigma\sqrt{2\pi}}$$

In our model, if A infects B, then in the absence of further information about the locations of the two individuals, or about the time at which A was infected, the probability that the transmission occurred at time T is given by the standard infectivity profile of equation 3.

$$P(T|S_A, \alpha, \beta, s) = \frac{e^{-(T-S_A+s)/\beta}(T - S_A + s)^{\alpha-1}\beta^{-\alpha}}{\Gamma(\alpha)}$$

For the purpose of generating simulations we elaborated on this model by decomposing the infectivity profile to be conditional on the time between A being infected and A becoming symptomatic. Where we suppose that A was infected at time zero, we have

$$P(T|S_A, \alpha, \beta, s) = \sum_{x=1}^{\infty} P(S_A = x|\mu, \sigma, X_0) P(T|S_A = x)$$

Where the first term within the sum is the time to symptom onset and the second term is an infectivity profile conditional on x. At this point, this distribution is unknown, except that, for obvious reasons, T is greater than or equal to x: Individual A cannot transmit the virus before being infected by the virus.

For the purpose of our simulations, we generated an approximate series of distributions, each having the form of an offset gamma distribution, equivalent to the original distribution, giving the expression

$$P(T|S_A, \alpha, \beta, s) = \sum_{x=1}^{\infty} P(S_A = x|\mu, \sigma, X_0) P(T|S_A, \alpha_x, \beta_x, x)$$

Where specifically

$$P(T|S_A, \alpha_x, \beta_x, s) = \frac{e^{-(T-S_A+x)/\beta_x}(T - S_A + x)^{\alpha_x - 1}{\beta_x}^{-\alpha_x}}{\Gamma(\alpha)}$$

We then optimised the coefficients α_x and β_x so as to minimise the RMS distance D between the two distributions:

$$D = \sqrt{\sum_{T=0}^{T=80} \left(P(T|S_A, \alpha, \beta, s) - \sum_{x=1}^{\infty} P(S_A = x|\mu, \sigma, X_0) P(T|S_A, \alpha_x, \beta_x, x) \right)^2}$$

A simple minimisation routine was implemented to perform this optimisation, terminating after 10^6 iterations. Supplementary Figure S9 shows the fit of our model to the original distribution, while our inferred parameters are shown in Table S3.

While we do not claim that our conditional distributions give a precise description of the reality of SARS-CoV-2 transmission, we obtain from this process a model which closely approximates the unconditional infectivity profile inferred from previous literature, while never producing the unrealistic outcome that an individual infects another without themselves being infected.

S4 Text: GISAID identifiers for sequences used in this study

Ward X: EPI_ISL_473479, EPI_ISL_473505, EPI_ISL_473478, EPI_ISL_473470, EPI_ISL_473475, EPI_ISL_473464, EPI_ISL_473467, EPI_ISL_473466, EPI_ISL_473465, EPI_ISL_473472, EPI_ISL_473471

Ward Y: EPI_ISL_425263, EPI_ISL_433686, EPI_ISL_444320, EPI_ISL_433740, EPI_ISL_444407, EPI_ISL_433479, EPI_ISL_433779, EPI_ISL_433481, EPI_ISL_448052, EPI_ISL_433492, EPI_ISL_433990

Real-time analysis: CAMB-1BC48E, CAMB-1BC460, CAMB-1BD669, CAMB-1BC336, CAMB-A98971, CAMB-1BB7B3, CAMB-1BD5F3, CAMB-1BB5E6, CAMB-A9AF04

Measurement error analysis: EPI_ISL_444341, EPI_ISL_434058, EPI_ISL_438599, EPI_ISL_425289, EPI_ISL_433900, EPI_ISL_425316, EPI_ISL_425424, EPI_ISL_433822, EPI_ISL_433820, EPI_ISL_425314, EPI_ISL_447952, EPI_ISL_433796, EPI_ISL_433814, EPI_ISL_433816, EPI_ISL_433666, EPI_ISL_425333, EPI_ISL_438723, EPI_ISL_433473, EPI_ISL_434042, EPI_ISL_425334, EPI_ISL_433978, EPI_ISL_438648, EPI_ISL_434020, EPI_ISL_444418, EPI_ISL_434059, EPI_ISL_438627, EPI_ISL_433671, EPI_ISL_433893, EPI_ISL_433827, EPI_ISL_434004, EPI_ISL_433775, EPI_ISL_434060, EPI_ISL_433938, EPI_ISL_444420, EPI_ISL_438714, EPI_ISL_448108, EPI_ISL_433895, EPI_ISL_438631, EPI_ISL_438594, EPI_ISL_433911, EPI_ISL_444425, EPI_ISL_425309, EPI_ISL_433899, EPI_ISL_433967, EPI_ISL_433681, EPI_ISL_425235, EPI_ISL_425259, EPI_ISL_425423, EPI_ISL_425252, EPI_ISL_425251, EPI_ISL_438650, EPI_ISL_425274, EPI_ISL_425427, EPI_ISL_425271, EPI_ISL_425270, EPI_ISL_425268, EPI_ISL_433673, EPI_ISL_433698, EPI_ISL_433675, EPI_ISL_433697, EPI_ISL_425453, EPI_ISL_433679, EPI_ISL_433677, EPI_ISL_433748, EPI_ISL_433784, EPI_ISL_438669, EPI_ISL_433672, EPI_ISL_434057, EPI_ISL_433737, EPI_ISL_438580, EPI_ISL_438673, EPI_ISL_433477, EPI_ISL_433727, EPI_ISL_433750, EPI_ISL_433752, EPI_ISL_433706, EPI_ISL_433707, EPI_ISL_433846, EPI_ISL_433721, EPI_ISL_438706, EPI_ISL_433732, EPI_ISL_444416, EPI_ISL_433806, EPI_ISL_434039, EPI_ISL_433966, EPI_ISL_433761, EPI_ISL_433768, EPI_ISL_433792, EPI_ISL_433873, EPI_ISL_433881, EPI_ISL_433868, EPI_ISL_444329, EPI_ISL_444408, EPI_ISL_433867, EPI_ISL_433878, EPI_ISL_433863, EPI_ISL_433467, EPI_ISL_433864, EPI_ISL_433888, EPI_ISL_444403, EPI_ISL_434023, EPI_ISL_433892, EPI_ISL_433907, EPI_ISL_433904, EPI_ISL_433906, EPI_ISL_438583, EPI_ISL_438647, EPI_ISL_444427, EPI_ISL_433909, EPI_ISL_433920, EPI_ISL_438721, EPI_ISL_444413, EPI_ISL_433921, EPI_ISL_433919, EPI_ISL_433980, EPI_ISL_433937, EPI_ISL_433993, EPI_ISL_433940, EPI_ISL_434031, EPI_ISL_438586, EPI_ISL_433466, EPI_ISL_444419, EPI_ISL_433944, EPI_ISL_433943, EPI_ISL_438651, EPI_ISL_433945, EPI_ISL_433969, EPI_ISL_438595, EPI_ISL_434006, EPI_ISL_433972, EPI_ISL_434030, EPI_ISL_433986, EPI_ISL_434061, EPI_ISL_433992, EPI_ISL_438668, EPI_ISL_438702, EPI_ISL_438649, EPI_ISL_444428, EPI_ISL_434034, EPI_ISL_438578, EPI_ISL_438660, EPI_ISL_434036, EPI_ISL_438643, EPI_ISL_444417, EPI_ISL_434015, EPI_ISL_433493, EPI_ISL_444316, EPI_ISL_434044, EPI_ISL_438662, EPI_ISL_438622, EPI_ISL_438596, EPI_ISL_434045, EPI_ISL_444374, EPI_ISL_434041, EPI_ISL_448052, EPI_ISL_433481, EPI_ISL_438632, EPI_ISL_433471, EPI_ISL_444412, EPI_ISL_433468, EPI_ISL_444369, EPI_ISL_433478, EPI_ISL_433475, EPI_ISL_438652, EPI_ISL_433476, EPI_ISL_438719, EPI_ISL_444326, EPI_ISL_447957,

EPI_ISL_444402, EPI_ISL_433474, EPI_ISL_444406, EPI_ISL_438670, EPI_ISL_444404, EPI_ISL_444376, EPI_ISL_452887, EPI_ISL_438582, EPI_ISL_444429, EPI_ISL_444411, EPI_ISL_447980, EPI_ISL_444422, EPI_ISL_444373, EPI_ISL_438672, EPI_ISL_444421, EPI_ISL_477785, EPI_ISL_456720, EPI_ISL_448012, EPI_ISL_453003, EPI_ISL_456719, EPI_ISL_438568, EPI_ISL_444324, EPI_ISL_444414, EPI_ISL_448110, EPI_ISL_438623, EPI_ISL_438588, EPI_ISL_438592, EPI_ISL_444339, EPI_ISL_438609, EPI_ISL_438646, EPI_ISL_438584, EPI_ISL_438728, EPI_ISL_438637, EPI_ISL_438591, EPI_ISL_438679, EPI_ISL_438671, EPI_ISL_438593, EPI_ISL_438658, EPI_ISL_438656, EPI_ISL_447948, EPI_ISL_438653, EPI_ISL_438645, EPI_ISL_438644, EPI_ISL_447974, EPI_ISL_438678, EPI_ISL_438674, EPI_ISL_438676, EPI_ISL_438699, EPI_ISL_448016, EPI_ISL_438680, EPI_ISL_447961, EPI_ISL_438701, EPI_ISL_438681, EPI_ISL_444371, EPI_ISL_447978, EPI_ISL_448014, EPI_ISL_444323, EPI_ISL_438697, EPI_ISL_448087, EPI_ISL_438737, EPI_ISL_452946, EPI_ISL_452962, EPI_ISL_444343, EPI_ISL_444361, EPI_ISL_456743, EPI_ISL_447977, EPI_ISL_452997, EPI_ISL_447955, EPI_ISL_447990, EPI_ISL_452922, EPI_ISL_453002, EPI_ISL_447994, EPI_ISL_456703, EPI_ISL_452973, EPI_ISL_452912, EPI_ISL_448017, EPI_ISL_448036, EPI_ISL_456686, EPI_ISL_448092, EPI_ISL_473460, EPI_ISL_448043, EPI_ISL_452911, EPI_ISL_452936, EPI_ISL_448050, EPI_ISL_456717, EPI_ISL_448106, EPI_ISL_473469, EPI_ISL_456724, EPI_ISL_448105, EPI_ISL_452935, EPI_ISL_456737, EPI_ISL_452868, EPI_ISL_448099, EPI_ISL_456741, EPI_ISL_452863, EPI_ISL_452963, EPI_ISL_461562, EPI_ISL_452939, EPI_ISL_452938, EPI_ISL_452951, EPI_ISL_456704, EPI_ISL_452994, EPI_ISL_456709, EPI_ISL_452989, EPI_ISL_452985, EPI_ISL_461558, EPI_ISL_452984, EPI_ISL_461573, EPI_ISL_473451, EPI_ISL_456708, EPI_ISL_456725, EPI_ISL_456728, EPI_ISL_456701, EPI_ISL_461585, EPI_ISL_456699, EPI_ISL_461561, EPI_ISL_456749, EPI_ISL_473484, EPI_ISL_473485, EPI_ISL_456742, EPI_ISL_461546, EPI_ISL_461587, EPI_ISL_461571, EPI_ISL_461554, EPI_ISL_473463, EPI_ISL_461565, EPI_ISL_461568, EPI_ISL_473501, EPI_ISL_473473
